# Machine learning prediction model for lateral lymph node metastasis in rectal cancer

**DOI:** 10.3389/fgstr.2025.1598686

**Published:** 2025-06-16

**Authors:** Longchun Dong, Shiyong Du, Hongjie Yang, Xipeng Zhang, Zhichun Zhang, Shuan Geng, Yuanda Zhou, Peng Li, Qingsheng Zeng, Yi Sun, Peishi Jiang

**Affiliations:** ^1^ Department of Radiology, Tianjin Union Medical Center, The First Affiliated Hospital of Nankai University, Tianjin, China; ^2^ Nankai University, Tianjin, China; ^3^ Department of Colorectal Surgery, Tianjin Union Medical Center, The First Affiliated Hospital of Nankai University, Tianjin, China; ^4^ The Institute of Translational Medicine, Tianjin Union Medical Center of Nankai University, Tianjin, China; ^5^ Tianjin Institute of Coloproctology, Tianjin Union Medical Center, Tianjin, China

**Keywords:** rectal neoplasms, lymphatic metastasis, prognosis, pathology, lateral lymph node dissection

## Abstract

**Background:**

The preoperative diagnosis of lateral lymph node metastasis presents a significant challenge. In this study, we aimed to predict the pathological characteristics of lateral lymph nodes in patients with rectal cancer using preoperative clinical information and to develop a logistic prediction model for lateral lymph node metastasis.

**Methods:**

A retrospective analysis of 143 patients who underwent total mesorectal excision (TME) and lateral lymph node dissection (LLND) at Tianjin Union Medical Center, from January 2017 to June 2024 was conducted. Patients were categorized into lateral lymph node metastasis and non-metastasis groups based on postoperative pathological findings. Basic information, tumor markers, and MRI reports were compared. Patients were segmented into training and validation sets at an 8:2 ratio. The R software was used to create a logistic prediction model and a nomogram.

**Results:**

This study included 66 pathologically positive and 77 pathologically negative lateral lymph node cases. Extramural vascular invasion (EMVI), MRI clinical N stage (MRI cN stage), and the number of enlarged lateral lymph nodes (NoELLN) were used to construct the logistic prediction model. The model achieved an accuracy of 0.62, sensitivity of 0.80, specificity of 0.43, and area under the curve (AUC) of 0.80 in predicting the pathological characteristics of lateral lymph nodes using the test dataset.

**Conclusion:**

EMVI, MRI cN stage, and NoELLN are significant predictive factors for predicting lateral lymph node pathology in patients with rectal cancer. These findings offer guidance for determining patient eligibility for LLND surgery.

## 1 Introduction

Lymph node metastases in rectal cancer mainly occur in mesenteric and lateral lymph nodes. Mesenteric lymph nodes are within the excision area of total mesorectal excision (TME). TME refers to the complete resection of the rectum, together with the mesorectum and the mesenteric lymph nodes in the mesenteric envelope. This surgical method is widely used and results in a substantial reduction in the local recurrence (LR) rate of rectal cancer after surgery (1). The conventional treatment strategies for lateral lymph node metastases include the following: 1) TME after neoadjuvant chemoradiotherapy (nCRT), 2) TME combined with lateral lymph node dissection (LLND), and 3) TME combined with LLND after nCRT. The nCRT treatment strategy is ineffective in the treatment of lateral lymph node metastases (2). Previous findings indicate that LLND can reduce the LR rate after rectal cancer surgery, and lateral lymph node dissection improves patient survival rates when performed accurately (3). Previous studies have reported pathologically positive rates of 27.9%, 37.0%, and 39.3% for lateral lymph nodes after LLND surgery (4–6). This implies that unnecessary dissection was performed in more than 60% of the cases. As a result, patients were subjected to the risks and side effects of a surgical procedure with no oncologic benefit. Understanding the treatment strategies and indications can be used to determine the eligibility of patients for the LLND procedure. Therefore, it is imperative to evaluate the status of the lateral lymph nodes before conducting surgery.

Previous studies have reported that machine learning-based diagnostic models have higher diagnostic efficiency than traditional imaging-based diagnostic methods in the diagnosis of lymph node metastasis in rectal cancer (7–9). In the present study, the clinical value of the logistic prediction model, based on the basic information of patients, tumor markers of the digestive system, and MRI image report information in the prediction of the pathological characteristics of lateral lymph nodes in patients with rectal cancer who have not received nCRT, was explored. The findings of this study provide a scientific reference for the development of personalized treatment approaches and a basis to improve the prognosis of patients with rectal cancer.

## 2 Materials and methods

### 2.1 Data retrieval

**Figure 1:**
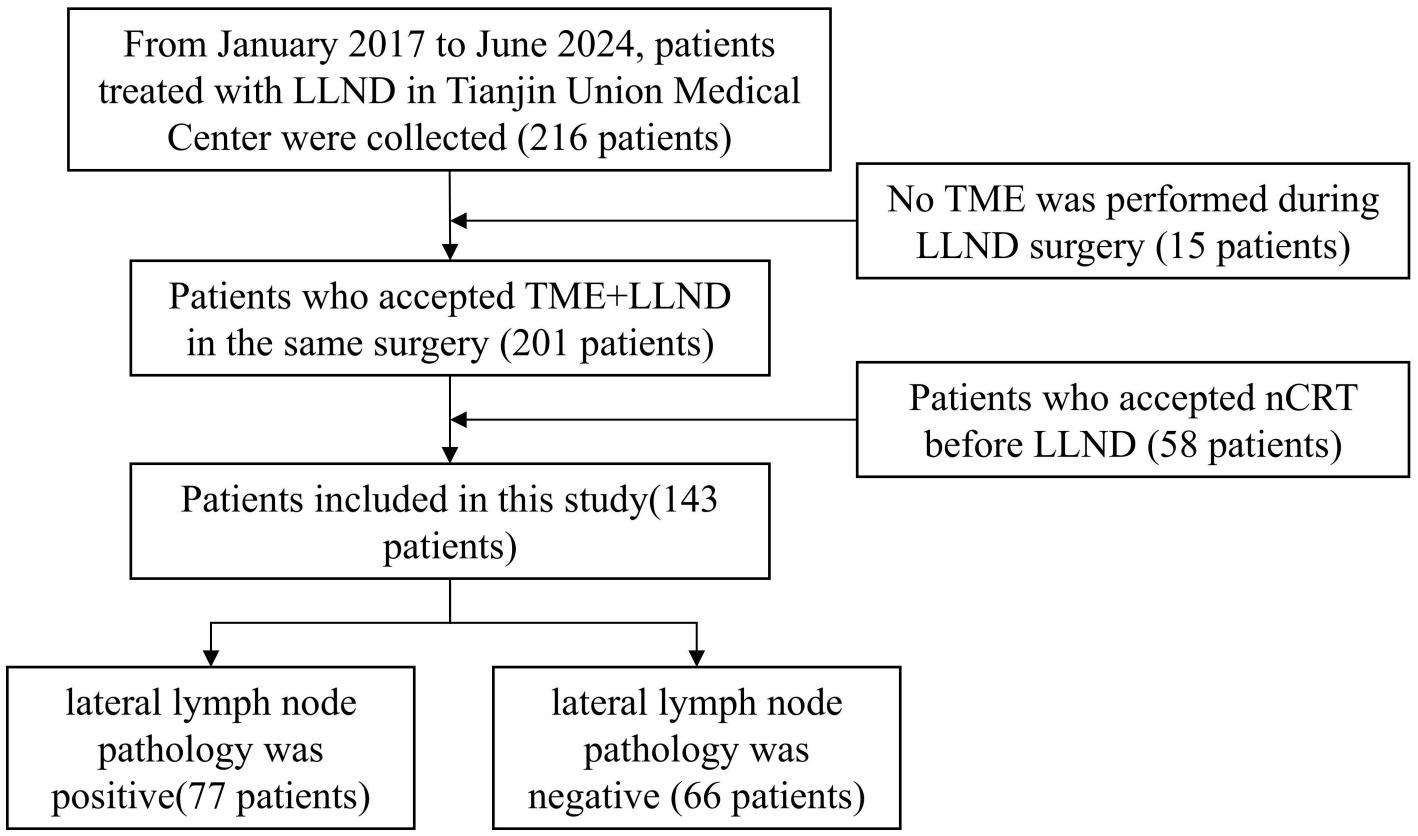
Flowchart showing the patient selection process. LLND, lateral lymph node dissection; nCRT, neoadjuvant chemoradiotherapy; TME, total mesorectal excision.

Patients who had undergone TME and LLND from January 2017 to June 2024 at Tianjin Union Medical Center were included in this study. The inclusion criteria were as follows: 1) patients diagnosed with rectal cancer by endoscopy biopsy; 2) patients who had undergone TME and LLND according to the *Protocol of Diagnosis and Treatment of Colorectal Cancer in China (2020 Edition)* and the *Expert Consensus on the Diagnosis and Treatment of Lateral Lymph Node Metastasis in Rectal Cancer in China (2019 Edition)*; 3) patients who had not received nCRT previously but had undergone TME and LLND; and 4) patients with pelvic MRI performed 2 weeks prior to surgery and lateral lymph nodes with a short-axis diameter exceeding 5 mm on the MRI, as assessed by the surgical team preoperatively. The exclusion criteria in this study were as follows: 1) patients with tumor invasion that required combined organ resection and 2) patients with unresectable distant metastases. Written informed consent was waived in this retrospective study. The study protocol was approved by Tianjin Union Medical Center’s Ethics Committee (Approval Nos. 2022-C23 and 2025-B81). **Figure 1** shows the inclusion and exclusion processes.

### 2.2 Collection of clinical data

Pelvic MRI and tumor marker detection of the digestive system were conducted 1 week prior to the surgery. The preoperative information collected included the following: 1) basic patient information: gender, age, height, and weight; 2) preoperative tumor markers of the digestive system: carcinoembryonic antigen (CEA) and carbohydrate antigen 19-9 (CA19-9); and (3) preoperative MRI image report information including the MRI clinical T stage (MRI cT stage), MRI clinical N stage (MRI cN stage), length of tumor, tumor-to-anal-margin distance, circumferential resection margin (CRM), extramural vascular invasion (EMVI), number of enlarged lateral lymph nodes (NoELLN), and number of enlarged mesorectal lymph nodes (NoEMLN). The criterion for enlarged lymph nodes was that the short diameter was greater than 5 mm, as determined by the radiologists who issued the MRI reports.

### 2.3 Assessment of lymph node metastasis

On preoperative MRI, a short‐axis diameter ≥5 mm of lateral pelvic lymph nodes is a key predictor of lateral metastasis and thus guides the decision to perform LLND (10). In this study, nodal involvement detected by MRI was assessed by the surgical team on T1-weighted turbo spin echo imaging in the axial plane and T2-weighted turbo spin echo imaging in the axial, coronal, and sagittal planes by evaluating both mesorectal lymph nodes (from the mesorectum to the course of the superior rectal artery) and lateral lymph nodes (internal iliac, external iliac, and obturator regions) for a short‐axis diameter ≥5 mm. Postoperative lymph node metastasis was then confirmed by pathologists on H&E-stained sections based on the presence of malignant cell morphology. Pelvic MRI was performed on a Siemens Magnetom Skyra 3.0 T or Philips Kbuitho 1.5 T scanner.

### 2.4 Statistical methods

All statistical analyses were performed using R (version 4.2.0) and Python (version 3.7) software. Missing value visualization was done with the VIM package in R (11). Missing values in continuous variables were imputed by predictive mean matching using the mice package in R (12). The *t*-test and standard Pearson chi-square test were conducted using R’s built-in stats package. Measurement data were expressed as the mean ± standard deviation (SD), and the independent sample *t*-test was used for comparisons between two groups of continuous data. Categorical data were expressed as *n* (%), and the *χ^2^* test was used for comparison between the groups. Variables with a *p*-value <0.1 were included in the multivariate analysis (13). The multivariate logistic regression method was used to construct a prediction model for the pathological characteristics of lateral lymph nodes in patients with rectal cancer after surgery using the scikit-learn package in Python (14). Subsequently, a nomogram was established using the rms package in R. Patient data were grouped into the training set and test set at a ratio of 8:2. The training set was used for the construction of a prediction model. The test set was used to determine the accuracy, sensitivity, and specificity of the model. A receiver operating characteristic (ROC) curve was generated, and the area under the curve (AUC) value was determined. All *p*-values were two-sided and *p <*0.05 was considered statistically significant.

## 3 Results

### 3.1 Basic patient information

**Figure 2:**
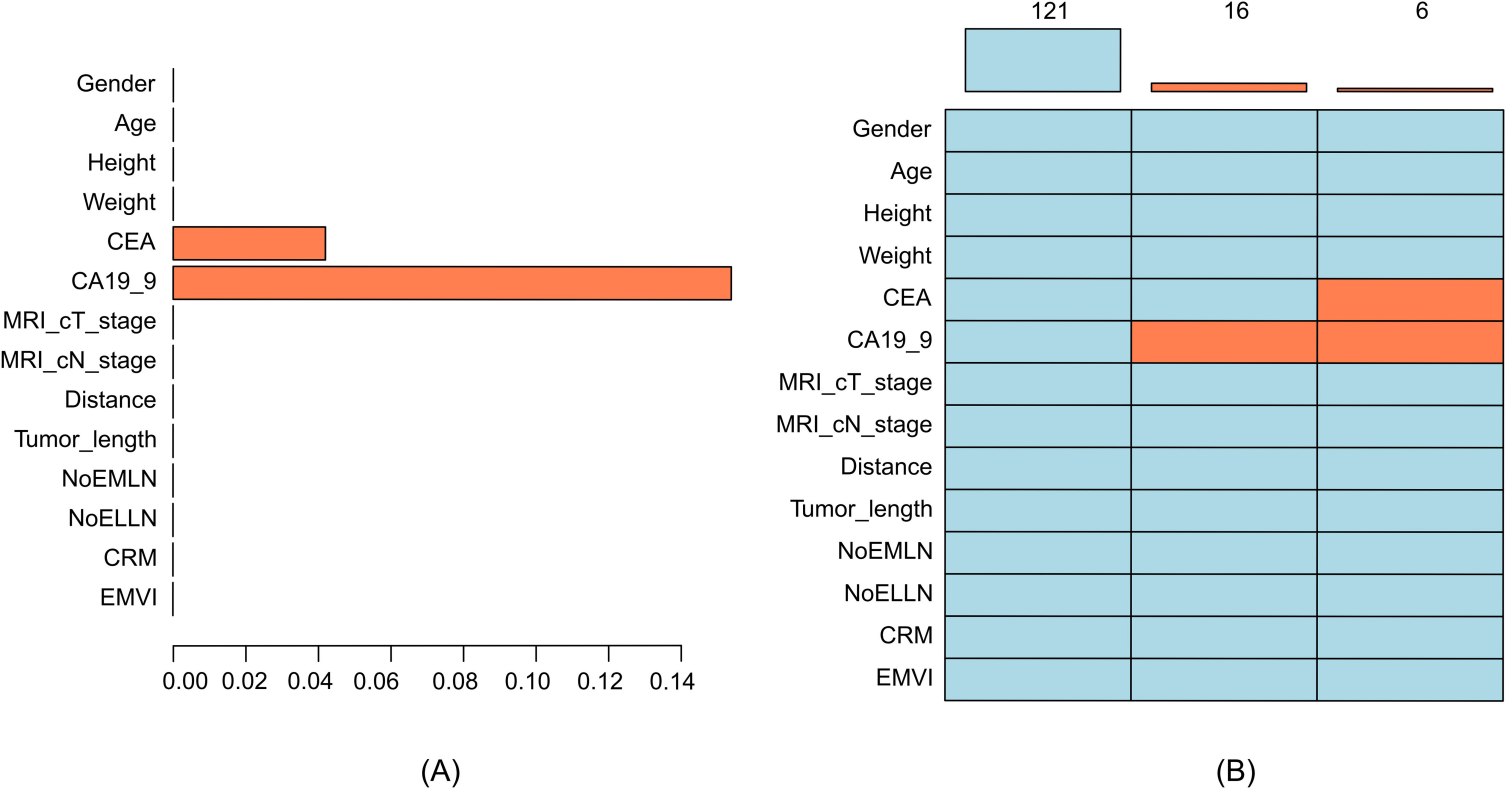
**(A)** Histogram of missing data, where the *X*-axis represents the proportion of missing values and the *Y*-axis represents the variable names. **(B)** Pattern of missing data, where each row represents a variable, and orange–red indicates missing values. CA19-9 was independently missing in 16 cases, and both CA19-9 and CEA were missing in 6 cases. CRM, circumferential resection margin; EMVI, extramural vascular invasion; CEA, carcinoembryonic antigen; CA19-9, carbohydrate antigen 19-9; NoELLN, number of enlarged lateral lymph nodes; NoEMLN, number of enlarged mesorectal lymph nodes.

A total of 143 patients who met the inclusion criteria were included in this study, and a total of 14 variables were evaluated in this study. The rate of missing data for each variable was less than 20% (**Figure 2**). The patients enrolled in the study included 49 women and 94 men. The age of the included patients ranged between 26 and 87 years, with an average age of 61.35 ± 12.02 years. In this study, 66 cases of positive lateral lymph nodes and 77 cases of negative lateral lymph nodes were observed after the postoperative pathological examination. Out of the 33 patients initially assessed by radiologists as having no lateral lymph nodes >5 mm, the surgical team identified enlarged lateral lymph nodes (>5 mm) on MRI preoperatively. After surgery, 5 of these patients were found to have pathologically positive lateral lymph nodes, while 28 cases were found to have negative lateral lymph nodes. The results significantly differed in EMVI (*p* = 0.008) and NoELLN (*p* = 0.001) between the lateral and the non-lateral lymph node metastasis groups (**Tables 1**, **2**).

**Table 1:** Categorical variables of patients in the lateral lymph node metastasis group and the non-lateral lymph node metastasis group.

Variable	Category	Non-metastasis group (*n* = 77)	Metastasis group (*n* = 66)	*χ* ^2^	*p*
Gender, *n* (%)	Female	24 (31.17)	25 (37.88)	0.71	0.399
	Male	53 (68.83)	41 (62.12)		
CRM, *n* (%)	−	36 (46.75)	24 (36.36)	1.58	0.209
	+	41 (53.25)	42 (63.64)		
EMVI, *n* (%)	−	52 (67.53)	30 (45.45)	7.08	0.008*
	+	25 (32.47)	36 (54.55)		
MRI cN stage, *n* (%)	mriN0	14 (18.18)	5 (7.58)	5.52	0.063
	mriN1	38 (49.35)	29 (43.94)		
	mriN2	25 (32.47)	32 (48.48)		
MRI cT stage, *n* (%)	mriT2	8 (10.39)	7 (10.61)	2.47	0.291
	mriT3	55 (71.43)	53 (80.30)		
	mriT4	14 (18.18)	6 (9.09)		

CRM, circumferential resection margin; EMVI, extramural vascular invasion; MRI cT stage, MRI clinical T stage; MRI cN stage, MRI clinical N stage.

*p < 0.05 (statistical significance).

**Table 2:** Continuous variables of patients in the lateral lymph node metastasis group and the non-lateral lymph node metastasis group.

Variable	Overall (*n* = 143)	Non-metastasis group (*n* = 77)	Metastasis group (*n* = 66)	*t*	*p*
Age, mean ± SD, years	61.10 ± 11.97	60.18 ± 10.92	62.17 ± 13.02	−0.98	0.326
Height, mean ± SD, cm	167.24 ± 7.85	167.68 ± 7.60	166.74 ± 8.10	0.70	0.482
Weight, mean ± SD, kg	68.43 ± 12.05	69.32 ± 12.31	67.38 ± 11.66	0.96	0.339
CEA, mean ± SD, ng/mL	33.11 ± 175.86	51.56 ± 237.66	11.59 ± 15.78	1.35	0.178
CA19-9, mean ± SD, ng/mL	63.82 ± 213.80	53.93 ± 191.06	75.36 ± 237.07	−0.59	0.553
Distance, mean ± SD, cm	4.52 ± 2.30	4.78 ± 2.31	4.22 ± 2.25	1.45	0.148
Tumor length, mean ± SD, cm	4.63 ± 1.89	4.41 ± 1.59	4.87 ± 2.15	−1.46	0.146
NoELLN, mean ± SD	1.26 ± 1.11	0.99 ± 1.05	1.58 ± 1.10	−3.24	0.001*
NoEMLN, mean ± SD	1.77 ± 1.83	1.53 ± 1.53	2.05 ± 2.09	−1.64	0.104

CEA, carcinoembryonic antigen; CA19-9, carbohydrate antigen 19-9; NoELLN, number of enlarged lateral lymph nodes; NoEMLN, number of enlarged mesorectal lymph nodes.

*p < 0.05 (statistical significance).

### 3.2 Logistic prediction model of lateral lymph node metastasis in rectal cancer

**Figure 3:**
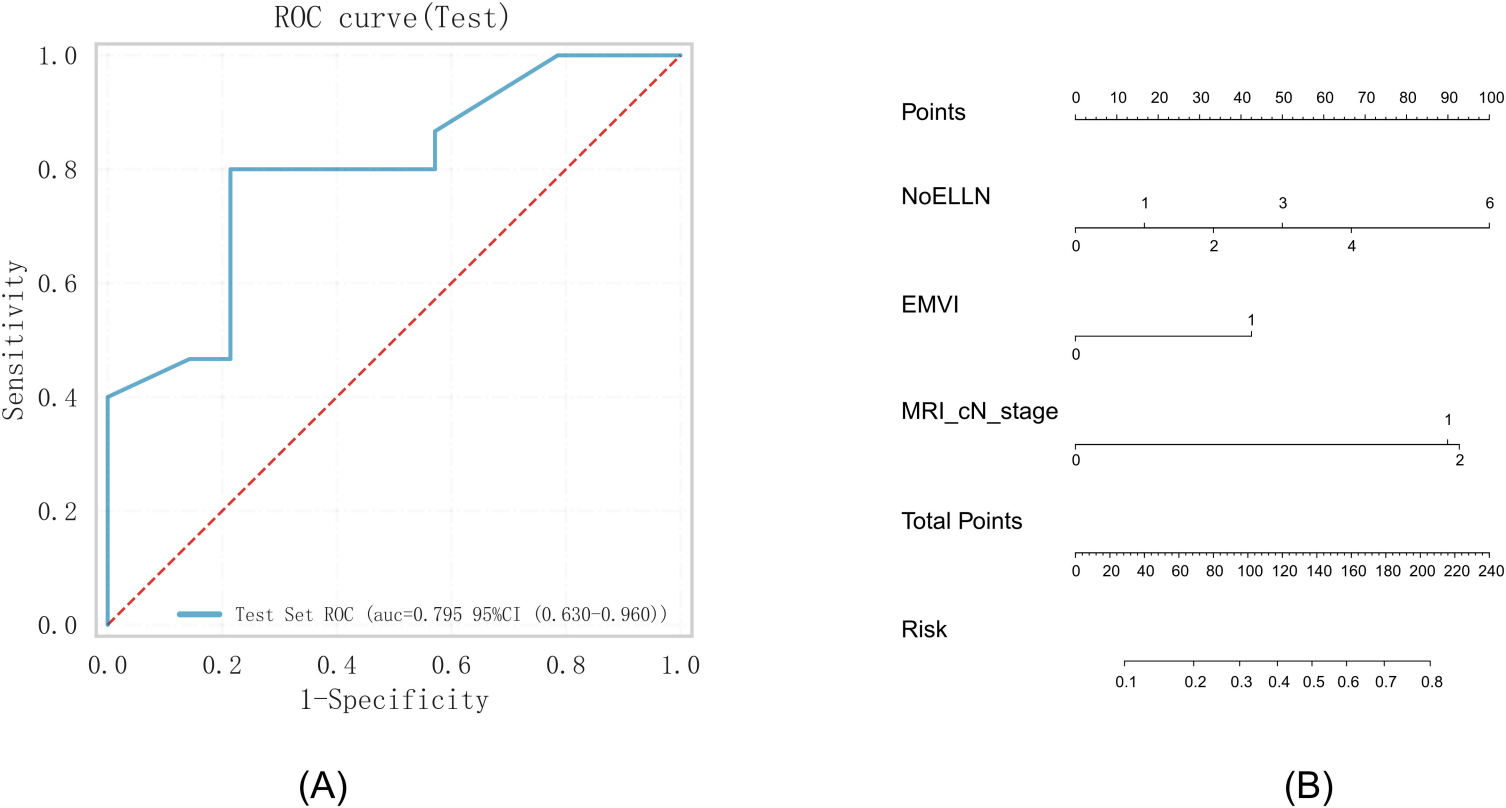
**(A)** ROC curve. **(B)** Nomogram based on the independent risk factors. ROC, operating characteristic curve; EMVI, extramural vascular invasion; AUC, area under the curve; NoELLN, number of enlarged lateral lymph nodes.

Three predictive factors for lateral lymph node metastases, namely, EMVI (*p* = 0.008), MRI cN stage (*p* = 0.063), and NoELLN (*p* = 0.001), were used to establish a prediction model. The logistic prediction model was built using a validation set comprising 114 patients. The pathological characteristics of the lateral lymph nodes in 29 patients in the test set were used to determine the accuracy, sensitivity, and specificity of the model. The accuracy of the model was 0.62, the sensitivity was 0.80, and the specificity was 0.43. The AUC of the ROC curve was 0.80 (0.63–0.96). These findings indicated a high prediction significance and accuracy of the model (**Figure 3**).

## 4 Discussion

The conventional treatment method for patients with suspected or confirmed lateral lymph node metastasis is the nCRT approach. Subsequently, LLND is performed based on whether the patient has a residual tumor in the lateral lymph nodes. TME and LLND were recommended for patients with suspected or confirmed lateral lymph node metastasis who declined nCRT (6). It is imperative to evaluate the status of the lateral lymph nodes before nCRT or surgery. Abe et al. (15) found that 77.8% of patients with lateral lymph nodes positive for pathology were EMVI-positive, whereas in patients with lateral lymph nodes negative for pathology, this proportion was only 36.7%, showing a statistically significant difference. In our study, we obtained similar results: 54.55% of patients with lateral lymph nodes positive for pathology were EMVI-positive, while in patients with lateral lymph nodes negative for pathology, this proportion was only 32.47%, showing a statistically significant difference. Previous studies have reported that the short-axis diameter of lateral lymph nodes on medical images is associated with a pathologically positive diagnosis (16, 17). Similar results were found in our research, where NoELLN was identified as a statistically significant variable between the lateral and non-lateral lymph node metastasis groups.

This study revealed a discrepancy between radiologists and the surgical team in identifying enlarged lateral lymph nodes >5 mm. Radiologists found no enlarged lateral lymph nodes in 33 patients, while the surgical team’s preoperative assessments identified them. This discrepancy likely stems from the surgical team’s stricter approach, driven by the serious implications of metastasis (18, 19). Kobayashi et al. (20) reported different optimal cutoff values for the long-axis diameter and short-axis diameter of lymph nodes in the diagnosis of mesenteric lymph node metastasis and lateral lymph node metastasis. The values were 6.5 mm and 5.7 mm, respectively, for the long-axis diameter and 9.0 mm and 6.0 mm, respectively, for the short-axis diameter. Fung et al. (21) concluded that a short-axis diameter of the lateral lymph node ≥7 mm indicates a diagnosis of lateral lymph node metastasis, and nCRT should be conducted. A short-axis diameter of the lymph node ≥4 mm after nCRT indicates the presence of a residual tumor, and TME and LLND should be conducted. Yamaoka et al. (17) reported that the optimal cutoff value of the short-axis diameter for the diagnosis of lateral lymph node metastasis in patients who had not received nCRT was 6.0 mm, whereas the value for patients who had received nCRT was 5.0 mm. Some scholars have proposed that the size of the lymph node before and after nCRT should be compared during the diagnosis of lateral lymph node metastasis after nCRT. Doctors and patients should consider the probability of a residual tumor if there is no notable shrinkage of the lymph node after nCRT, especially with shrinkage of ≤33% to 60% (22). These findings highlight the necessity of distinct diagnostic criteria for lymph nodes in diagnosing lateral lymph node metastasis in patients with rectal cancer, differentiating between those who have not undergone nCRT and those who have received the treatment.

Imaging test procedures for the examination of lymph node metastasis in rectal cancer include MRI, CT, and intrarectal ultrasound. These procedures have low image sensitivity and specificity in assessing the properties of lymph nodes (23–25). Therefore, researchers are currently exploring the use of machine learning methods to assess lymph node metastasis in rectal cancer. Currently, the pathological characteristics of lymph nodes are often used as the dataset for machine learning without distinguishing between mesenteric and lateral lymph nodes. Therefore, the results obtained are not applicable for determining the eligibility of the patient for nCRT or LLND treatment (26). Nakanishi et al. (27) used the pathological characteristics of lateral lymph nodes as the dataset for machine learning to build a machine learning diagnostic model. Patients who had undergone nCRT, TME, and LLND were included in the study. The AUC of the model using the validation set was 0.91, indicating that the diagnostic model was effective in predicting the status of lateral lymph nodes in patients after nCRT. Studies have not been conducted on the construction of machine learning-based prediction models for the pathological characteristics of lateral lymph nodes in patients with rectal cancer who have not received nCRT. In this study, clinical data from patients who underwent TME and LLND were used as the dataset for the construction of a prediction model. The AUC of 0.80 (95% CI: 0.63–0.96) indicates that the machine learning-based model can accurately predict the status of the lateral lymph node in patients with rectal cancer who have not received nCRT; however, given the wide confidence interval, the model’s performance is associated with a certain degree of uncertainty. The results from the model provide a reference for determining whether the patient is eligible for nCRT or LLND. Our retrospective, single-center cohort of 143 patients may introduce selection bias and limit external validity. Nevertheless, these results provide a basis for multicenter studies to confirm our model’s diagnostic performance across diverse populations. An in-depth exploration of the specific mechanisms, dictating how these clinical risk factors influence the pathological characteristics of lateral lymph nodes, is an intriguing area for future research.

## 5 Conclusion

This study suggests that EMVI, MRI cN stage, and NoELLN could be potential determinants when assessing the suitability of patients with rectal cancer who have not yet undergone nCRT for either LLND surgery or nCRT in a clinical setting. These findings provide a reference for predicting the pathological characteristics of lateral lymph nodes after surgery.

## Data Availability

The original contributions presented in the study are included in the article. Further inquiries can be directed to the corresponding authors.
